# Polymorphisms in *FTO* and *MAF* Genes and Birth Weight, BMI, Ponderal Index, Weight Gain in a Large Cohort of Infants with a Birth Weight below 1500 Grams

**DOI:** 10.1371/journal.pone.0066331

**Published:** 2013-06-26

**Authors:** Sebastian Haller, Juliane Spiegler, Claudia Hemmelmann, Helmut Küster, Matthias Vochem, Jens Möller, Dirk Müller, Angela Kribs, Thomas Hoehn, Christoph Härtel, Egbert Herting, Wolfgang Göpel, the German Neonatal Network

**Affiliations:** 1 Department of Pediatrics, University of Lübeck, Lübeck, Germany; 2 Institute of Medical Biometry and Statistics, University of Lübeck, Lübeck, Germany; 3 Department of Pediatrics, University of Göttingen, Göttingen, Germany; 4 Olgahospital Stuttgart, Stuttgart, Germany; 5 Children's Hospital Saarbrücken, Saarbrücken, Germany; 6 Children's Hospital Kassel, Kassel, Germany; 7 Department of Pediatrics, University of Cologne, Cologne, Germany; 8 Department of Pediatrics, University of Düsseldorf, Düsseldorf, Germany; Vanderbilt University Medical Center, United States of America

## Abstract

**Background:**

The *FTO* gene, located on chromosome 16q12.2, and the *MAF* gene, located on chromosome 16q22-23, were identified as genes harboring common variants with an impact on obesity predisposition. We studied the association of common variants with birth weight, gain of body weight, body mass index (BMI), Ponderal index and relevant neonatal outcomes in a large German cohort of infants with a birth weight below 1500 grams.

**Methods:**

The single nucleotide polymorphisms rs9939609 (*FTO* gene) and rs1424233 (*MAF* gene) were genotyped using allelic discrimination assays in a prospective multicenter cohort study conducted in 15 neonatal intensive care units in Germany from September 2003 until January 2008. DNA samples were extracted from buccal swabs according to standard protocols.

**Results:**

1946 infants were successfully genotyped at *FTO* and 2149 infants at *MAF*. Allele frequencies were not significantly different from other European cohorts. The polymorphisms were in Hardy-Weinberg equilibrium. The polymorphisms did not show associations with birth weight, BMI and Ponderal Index at discharge, and weight gain, neither testing for a dominant, additive nor for a recessive model.

**Discussion:**

Since an association of the polymorphisms with weight gain has been demonstrated in multiple populations, the lack of association in a population of preterm infants with regular tube feeding after birth and highly controlled feeding volumes provides evidence for the hypothesis that these polymorphisms affect food intake behavior and hunger rather than metabolism and energy consumption.

## Introduction

Obesity is a major cause of morbidity and mortality, associated with an increased risk of type 2 diabetes mellitus, heart disease, metabolic syndrome, hypertension, stroke and certain forms of cancer [1]. Although twin and adoption studies [2] have suggested that BMI is highly heritable, progress in identifying specific variants involved in regulating body weight has largely been confined to rare mutations causing severe, monogenic early-onset forms of obesity [3]. Recently, genome-wide association studies (GWAs) have identified polymorphisms in or close to the *FTO* and *MAF* gene to be associated with obesity [4], [5], [6].

The *FTO* gene is located on chromosome 16q12.2. It is harboring common variants with an impact on obesity predisposition and fat mass at a population level [4], [5], [6], [7]. The rs9939609 *FTO* (T = >A) has been associated with BMI in numerous studies, altering the body weight of homozygous adults about 3 kilograms [4]. An association with an increased risk of childhood obesity was also demonstrated by Frayling *et al.* [4].

Meyre *et al.* have recently published a GWAs in which they identified a novel locus, with rs1424233 being the lead SNP, which is associated with obesity in Europeans [5]. The *MAF* gene is mapped about 48 kb away from rs1424233. The ubiquitously expressed c-MAF transcription factor is involved in developmental and cellular differentiation processes, notably of the immune system [8], adipose tissue [9], and pancreas [10].

Despite these associations, the mechanisms by which these polymorphisms affect the predisposition on obesity are not yet understood. Whether they might influence weight gain by alteration of food uptake, energy consumption, or metabolism is still subject to research [2], [11], [12], [13], [14].

Very low birth weight (VLBW)-infants are defined by birth before 37 weeks of gestation (the normal time of pregnancy is 40 weeks) and a birth weight below 1500 grams. About 1% of all newborns are VLBW-infants. They are furthermore characterized by the following conditions: 1. They are closely monitored since all individuals stay in hospital from birth on. 2. They are fed similarly tolerating only small deviations of the recommended amount of calories per kilogram (if infants are not drinking the recommended amount of milk they are fed by gastric tubes). 3. They are kept in warm and moist surrounding (e.g. incubators) to minimize energy consumption during their stay in hospital.

These special conditions may provide evidence for or against an impact of the polymorphisms on energy consumption or metabolism.

The objective of our study was to search for an association of *FTO* and *MAF* polymorphisms with birth weight, gain of weight, BMI, Ponderal index and relevant neonatal outcomes in a large German birth cohort of VLBW-infants.

## Materials and Methods

### Ethics Statement

The study was approved by the ethics committee of the University of Lübeck (approval number 08-022) and local ethics committees of all participating centers (Childrens Hospital Kassel; University of Leipzig; Kinderkrankenhaus auf der Bult, Hannover; Altonaer Kinderkrankenhaus, Hamburg; Childrens Hospital Aschaffenburg; University of Düsseldorf; St. Elisabeth Hospital, University of Bochum; University of Greifswald; University of Cologne; Olgahospital, Stuttgart; University of Regensburg; Childrens Hospital Saarbrücken; Childrens Hospital Eutin; University of Kiel; all in Germany). Parents gave written informed consent to all parts of the study.

### Study Population

The study population has been previously described in detail [15], [16]. Briefly, a prospective multicenter cohort study to investigate the influence of genetic variants involved in premature birth and diseases of VLBW-infants was performed in 15 neonatal intensive care units (NICUs) in Germany from September 2003 until January 2008 (inclusion criteria: birth weight <1500 g, gestational age ≥22+0 to ≤36+6 weeks; exclusion criteria: lethal malformations, e.g., trisomy 13, or trisomy 18). Maternal ethnicity was categorized as “Germany”, “other European descent”, “Turkey and Northern Africa”, “Sub-Sahara-Africa”, “Asia” and “Other”.

DNA samples were obtained by buccal swabs. Antenatal and postnatal treatment and outcome data were recorded by participating centers on standardized data sheets. A physician trained in neonatology evaluated the data quality by on-site monitoring every 6 months.

### Assessment of Phenotypes

Birth weight, body-length and head circumference at birth were available of all infants. Length of hospital stay until discharge or until death were recorded in days as well as body measurements at discharge or death. Hypertrophy (higher than expected weight compared to normal range values of the according gestational age) and hypotrophy (lower than expected weight compared to normal references values of the according gestational age) were calculated stratifying the population by gestational age and allocating each stratum minimum or maximum weight according to the Fenton scale [17]. These dichotomous variables indicate if the birth weight is >97th percentile, >90th percentile or <10th percentile when compared with the reference population and are estimates of prenatal growth. Furthermore, the Ponderal index at discharge (weight at discharge in kg/(body-length in meter) cubed) and BMI at discharge (weight at discharge in kg/m^2^) were calculated. Mean weight gain per day until discharge and mean gain of body length were also calculated for analysis.

### Genotyping

DNA samples were extracted from buccal swabs according to standard protocols. The single nucleotide polymorphism (SNP) rs1424233 from the *MAF* gene and rs9939609 from the *FTO* gene were genotyped using an allelic discrimination assay (Custom TaqMan MGB, Applied Biosystems, Foster City, USA). The sequences of the polymorphisms and the assay components have been described previously [4], [5].

### Statistical analysis

For dichotomous variables, such as birth weight >97th percentile, association was tested using the Wald test from the logistic regression for additive, dominant, and recessive genetic models. For continuous outcome variables, such as mean weight gain per day, linear regression was performed using additive, dominant, and recessive genetic models.

Both SNPs were investigated for compatibility with Hardy-Weinberg equilibrium using relative excess heterozygosity using the nominal 5% test level [18].

Statistical analyses were performed with SAS (version 9.2; SAS Institute Inc., Cary, USA) and SPSS for Windows (version 17.0; SPSS Inc., Chicago, USA).

## Results

2488 VLBW- infants participated in the study. There were 1261 male study participants and 1227 female participants. Sixty children died before discharge from hospital. Clinical data are given in table 1. Genotyping was successful in 78.2% (n = 1946) infants for rs9939609 and 86.4% (n = 2149) for rs1424233. Genotype frequencies were for rs9939609: TT 32.4%, AT 49.4%, and AA 18.2%, and for rs1424233; TT 25.7%, TC 50.5% and CC 23.8%. The *FTO*-A-allele frequency was 0.43 and the MAF-C-allele frequency 0.49. The distribution of the variants was in Hardy-Weinberg equilibrium (rs9939609: ω = 1.01 (90% confidence interval (CI) (0.94, 1.10)); rs1424233; ω = 1.02 (90% CI (0.95, 1.09))). Only 3.3% of the study population had a mother of non European descent.

**Table 1:** Hypertrophy and hypotrophy and clinical data in German vlbw infant population.

		*Gender*		
		male	female	total
**Birth weight <10th percentile** (Mean ± SD, gram)	no	n = 993 (40.1%) 1122±255	n = 895 (36.1%) 1096±274	n = 1888 (76.2%) 1110±265
	yes	n = 263 (10.6%) 977±341	n = 328 (13.2%) 950±353	n = 591 (23.8%) 962±348
**Birth weight >90th percentile** (Mean ± SD, gram)	no	n = 1221 (49.1%) 1090±282	n = 1204 (48.5%) 1056±305	n = 2425 (97.6%) 1073±294
	yes	n = 38 (1.5%) 1180±264	n = 23 (0.9%) 1168±271	n = 61 (2.4%) 1176±265
**Birth weight >97th percentile** (Mean ± SD, gram)	no	n = 1254 (50.4%) 1092±281	n = 1224 (49.3%) 1058±305	n = 2478 (99.7%) 1075±294
	yes	n = 5 (0.2%) 1305±236	n = 3 (0.1%) 1075±344	n = 8 (0.3%) 1219±282
**Mean gestational age (SD)**		28+5 (2.6)	29+0 (2.8)	28+6 (2.7)
**Mean birth weight in g (SD)**		1092 (281.5)	1058 (304.8)	1075 (293.7)
**Mean length at birth in cm (SD)**		36.8 (3.6)	36.8 (3.9)	36.8 (3.7)
**Mean head circumference in cm at birth (SD)**		26.0 (2.4)	25.7 (2.6)	25.9 (2.5)
**Study population total**		n = 1261 (51%)	n = 1227 (49%)	n = 2488 (100%)

### Birth weight

Associations according to mean birth weight by genotype are displayed in Table 2. Neither testing for trend nor testing dominant or recessive models showed significant differences. Logistic regression for groups hypertrophy (>97^th^ percentile and >90^th^ percentile) and hypotrophy (<10^th^ percentile) with adjustment for confounding by gender, multiples, year of birth, maternal ethnicity, and treating NICU did not reveal significant findings.

**Table 2:** Mean outcome distribution according to genotype and phenotype.

		birth weight (mean in g)	body-length at birth (mean in cm)	head circum-ference at birth (mean in cm)	full enteral feeding (mean in days)	weight gain (mean in g/d)	weight at discharge (mean in g)	BMI at discharge (mean)	length of hospital stay (mean days)
***FTO***	TT	1088	37.6	26	16	21.1	2577	12.1	71.3
	AT	1070	36.6	25.84	16	21.2	2531	12.1	71.2
	AA	1069	36.4	25.8	17	21.0	2586	12.3	73.5
***MAF***	TT	1083	36.7	25.82	15	21.1	2552	12.1	70.1
	TC	1066	36.6	25.8	17	20.9	2553	12.1	72.9
	CC	1090	36.7	26.0	15	21.5	2535	12.1	70.2
**NEC** *	no	1083	36.7	25.9	15	21.1	2543	12.1	70.4
	yes	832	33.7	23.9	44	16.8	2693	12.4	113.4
**Gender**	male	1092	36.8	26.0	16	21.3	2608	12.2	72.7
	female	1059	36.5	25.7	16	20.6	2484	12.0	70.5

* Surgery for necrotizing enterocolitis.

### Weight gain

Association analyses using linear regression for mean weight gain per day until discharge from hospital, mean gain of length, Ponderal index, and BMI did not reveal significant associations. Even adjustments for gender, multiples (twins, triplets and quadruplets), year of birth, APGAR in minute 10, umbilical-cord-artery-pH, birth in a level 1 neonatal intensive care unit, sepsis, surgical procedures, and treating NICU did not reveal associations with p<0.05. For these analysis VLBW-infants with fatal outcome were excluded (n = 60) to reduce variance until discharge.

Furthermore, none of the polymorphisms showed any influence on birth weight or weight gain if compared to established clinical factors such as gender or surgical treatment of necrotizing enterocolitis (table 2).

## Discussion

We could neither find an association of the rs9939609 or rs1424233 with birth weight nor weight gain in VLBW-infants. These findings support the hypothesis that the polymorphisms have no impact on energy consumption and metabolism but rather on food uptake behavior.

The *FTO* and *MAF* genes have been recently described to be major candidate genes for human obesity [4], [5], and these results have been replicated in multiple populations [6], [7]. To determine the impact of these polymorphisms on birth weight and neonatal weight gain we conducted this analysis. *FTO* and *MAF* allele-frequencies in our cohort were similar to those reported in other populations [4]. However, the prevalence of *FTO* and *MAF* SNPs was not associated with hypertrophy or hypotrophy in our study. Thus, we found no evidence for a relevance of these polymorphisms in intrauterine growth. These results are in line with the study by Frayling *et al.* who found no association of rs9939609 with birth weight in children from the UK [4].

As published previously, twin analysis support a strong genetic influence on postnatal growth in preterm infants [14]. VLBW-infants seem to be an excellent study collective for analysis on genetic influences on metabolism because of rapid growth and weight gain and accurate monitoring of body measures. Mean length of stay in hospital was about 72 days in our population. Throughout the study period the preterm infants remained in a controlled surrounding, where food intake was based on medical standards and variations in energy consumption were very limited. Neonatal weight gain did not correspond with prevalence of the polymorphisms tested even though we investigated several phenotypes of interest, including Ponderal index, BMI, and growth of length and gain of body weight per day, with and without adjustments for possible confounders.

In several cohorts association of the polymorphisms was found with obesity in children. In a small population Lopez-Bermejo *et al.* found an association of the *FTO*-polymorphism with gain of weight during the first two weeks of life in term born children [12]. Whether inconsistency of these results compared to ours are due to the small sample size of their population and lack of multiple testing, or caused by differences in populations will have to be investigated in future studies. The allele frequencies of *FTO* and *MAF* polymorphisms in our cohort did not differ significantly from other large populations [4], [5]. Rzehak *et al.* found no association of *FTO*-variants with birth weight in the GINI and LISA birth-cohort and could show that significant association developed over time in their population at the age of 4 years [19]. Furthermore, in the large Generation R Study, the FTO polymorphism was not associated with BMI in preschool children, which would be consistent with our results [20].

Both polymorphisms were neither associated with birth weight nor with growth of neonates. It may therefore be hypothesized that the SNPs rs9939609 and rs1424233 from the *FTO* and *MAF* genes do have a stronger impact on food intake behavior and hunger, than on metabolism and energy consumption. This supported by Wardle *et al.* who found not only a significant association of the rs9939609 AA genotypes with reduced satiety responsiveness scores, but also a mediating effect through satiety responsiveness on the association with increased adiposity [11]. Cecil *et al.* concluded from their analysis in a large cohort that *FTO* variants had impact on food intake and a preference for energy-dense foods [13].

Our study has several limitations. Due to our study design, we were not able to measure hunger and food intake directly. Furthermore, longitudinal assessment was limited until discharge. Therefore, we were not able to measure the combined long-time effects of metabolic programming (e.g. due to intrauterine growth restriction) and genotypes associated with obesity. This will be an important aim of the ongoing longitudinal GNN-study.

In summary, our negative results provide further evidence that neither rs9939609 from the *FTO* gene nor rs1424233 from the *MAF* gene influence metabolism and energy consumption, but might be of importance for food uptake behavior. Further research on the pathophysiologic effects of polymorphisms in these genes is needed to reveal the origin of association with obesity.
